# Molecular determination of O25b/ST131 clone type among extended spectrum β-lactamases production *Escherichia coli* recovering from urinary tract infection isolates

**DOI:** 10.1186/s12941-022-00526-2

**Published:** 2022-08-04

**Authors:** Amin Khoshbayan, Rezvan Golmoradi Zadeh, Majid Taati Moghadam, Shiva Mirkalantari, Atieh Darbandi

**Affiliations:** 1 Department of Microbiology, School of Medicine, Iran University of Medical Sciences, Tehran, Iran; 2 Antimicrobial Resistance Research Center, Institute of Immunology and Infectious Diseases, Iran University of Medical Sciences, Tehran, Iran

**Keywords:** *Escherichia coli*, ST131, ESBL, O25b/ST131, Iran

## Abstract

**Background:**

*Escherichia coli (E. coli)* O25b/ST131 clone causes urinary tract infection (UTI) and is associated with a broad spectrum of other infections, such as intra-abdominal and soft tissue infections, that can be affecting bloodstream infections. Therefore, since O25b/ST131 has been reported in several studies from Iran, in the current study, we have investigated the molecular characteristics, typing, and biofilm formation of O25b/ST131 clone type *E. coli* collected from UTI specimens.

**Methods:**

A total of 173 *E. coli* isolates from UTI were collected. The susceptibility to all fourth generations of cephalosporins (cefazolin, cefuroxime, ceftriaxone, cefotaxime, ceftazidime, cefepime) and ampicillin, ampicillin-sulbactam and aztreonam was determined. Class A ESBLs, class D ESBL and the presence of *pabB* gene screenings to detect of O25b/ST131 clone type were performed by using of PCR. Biofilm formation was compared between O25b/ST131 isolates and non-O25b/ST131 isolates. Finally, ERIC-PCR was used for typing of ESBL positive isolates.

**Results:**

Ninety-four ESBL positive were detected of which 79 of them were O25b/ST131. Antimicrobial susceptibility test data showed that most antibiotics had a higher rate of resistance in isolates of the O25b/ST131 clonal type. Biofilm formation showed that there was a weak association between O25b/ST131 clone type isolates and the level of the biofilm formation. ERIC-PCR results showed that *E. coli* isolates were genetically diverse and classified into 14 groups.

**Conclusion:**

Our results demonstrated the importance and high prevalence of *E. coli* O25b/ST131 among UTI isolates with the ability to spread fast and disseminate antibiotic resistance genes.

## Introduction

Uropathogenic *Escherichia coli* (UPEC) strains associated with a high incidence of community-acquired and hospital-acquired UTIs [1]. There are 150 million UTIs worldwide each year and drug-resistant infections typically require more complex treatment regimens and are more likely to occur if treatment is unsuccessful [2–4]. Furthermore, 70–95% of community-acquired UTIs are caused by UPEC which is the second most common infection in the community [4, 5]. The UTIs is the main cause of *E.coli* bloodstream infections leading, to 40,000 deaths from sepsis every year in the US [2, 6]. Moreover, *E. coli*, which especially causes extraintestinal infections, becomes resistant to every class of antibiotics used to treat such infections [7]. Unfortunately, the capacity of UPEC to obtain multiple drug resistance, particularly board-spectrum β-lactamases, may impede the therapeutic control of infections [1]. The clonal extension is an essential factor related to the diffusion of extended-spectrum beta-lactamases (ESBLs) producing *E. coli* isolates, mainly giving rise to the spread of multidrug-resistant (MDR) strains [8]. The CTX-M-type beta-lactamase enzyme, especially CTX-M-15, is the predominant ESBL, and is often found in *E. coli* sequence type 131 (ST131) [9]. *E. coli* ST131 was first discovered in 2008 based on the sequences inside seven *E. coli* housekeeping genes described as Multilocus Sequence Typing (MLST). According to MLST and molecular methods, such as PCR, studies have shown that the ST131 clone is an important human pathogen worldwide [10]. The *E. coli* ST131 clone causes UTI and is associated with a wide spectrum of other infections, such as bloodstream, soft tissue infections, and intra-abdominal, as well as epididymal-orchitis, meningitis, and septic shock [11]. In addition, *E.coli* forms a biofilm that is associated with the pathogenesis of diarrheagenic *E. coli* [12]. Biofilm formation by extraintestinal pathogenic *E. coli* (ExPEC) was largely observed in UPEC [13]. Therefore, as the few studies reported O25b/ST131 from Iran and the purpose of this study is to investigate the genetic characteristics, types, and biofilm formation methods of *E. coli* in O25b/ST131 clones to determine the extent of resistance and distribution of the most resistant clone.

## Methods and materials

### Bacterial strains

A total of 173 clinical isolates were collected from two selective hospitals in Tehran. The 94 ESBLs isolates were selected among them which, recovered from 33 male and 61 female. These isolates were collected from both outpatients and hospitalized patients over a period of 9 months from October 2018 to June 2019. In addition, all isolates recovered from urine and one strain per patient was investigated.

### Isolation and identification of E. coli isolates

All strains were isolated on MacConkey’s agar (Conda lab, Spain) and genotypically confirmed by amplification of the 16S rDNA gene by using ECO primers (Table 1) [14]. The Metabion (Germany) primers were used in this study.

**Table 1:** Nucleotide sequences of primers used in this study

Primer name	Sequence (5ʹ to 3ʹ)	Size of product (bp)	References
ECO-1	GACCTCGGTTTAGTTCACAGA	585	[14]
ECO-2	CACACGCTGACGCTGACCA		
TEM-F	GAGTATTCAACATTTCCGTGTC	848	[24]
TEM-R	TAATCAGTGAGGCACCTATCTC		
SHV-F	AAGATCCACTATCGCCAGCAG	231	[25]
SHV-R	ATTCAGTTCCGTTTCCCAGCGG		
CTX-M group 1-F	TTAGGAAGTGTGCCGCTGTA	688	[26]
CTX-M group 1-R	CGATATCGTTGGTGGTGCCAT		
CTX-M group 2-F	CGTTAACGGCACGATGAC	404	[26]
CTX-M group 2-R	CGATATCGTTGGTGGTGCCAT		
CTX-M group 8-F	TCGCGTTAAGCGGATGATGC	666	[27]
CTX-M group 8-R	AACCCACGATGTGGGTAGC		
CTX-M group 9-F	TCAAGCCTGCCGATCTGGT	561	[26]
CTX-M group 9-R	TGATTCTCGCCGCTGAAG		
CTX-M group 15-F	TTTCCCCATTCCGTTTCCGC	925	[28]
CTX-M group 15-R	TTCGTATCTTCCAGAATAAG		
CTX-M group 25-F	GCACGATGACATTCGGG	327	[27]
CTX-M group 25-R	AACCCACGATGTGGGTAGC		
GES-F	GCTTCATTCACGCACTATT	323	[29]
GES-R	CGATGCTAGAAACCGCTC		
PER-F	GCTCCGATAATGAAAGCGT	520	[30]
PER-R	TTCGGCTTGACTCGGCTGA		
VEB-F	CATTTCCCGATGCAAAGCGT	648	[30]
VEB-R	CGAAGTTTCTTTGGACTCTG		
OXA-10-F	TCAACAAATCGCCAGAGAAG	277	[31]
OXA-10-R	TCCCACACCAGAAAAACCA		
PABB-F	TCCAGCAGGTGCTGGATCGT	347	[18]
PABB-R	GCGAAATTTTTCGCCGTACTGT		
ERIC-F	ATGTAAGCTCCTGGGGATTCAC		[32]
ERIC-R	AAGTAAGTGACTGGGGTGAGCG		

### ESBL-confirmatory testing

The antibiotic disks BD (USA) and Mast (UK) were used to determining susceptibility profiles using the disk diffusion method. Furthermore, by using cefotaxime and ceftazidime with and without clavulanic acid disks determination of the ESBLs isolates was performed in accordance with Clinical and Laboratory Standards Institute (CLSI, 2020) guidelines. Increasing of  ≥ 5-mm in zone diameter of the ceftazidime-clavulanic acid compared to the zone diameter of ceftazidime considered as ESBLs [15, 16].

### Antimicrobial susceptibility testing

We also performed additional susceptibility testing on positive ESBL isolates using the following disks: cefazolin (30 μg), cefuroxime (30 μg), ceftriaxone (30 μg), cefepime (30 μg), aztreonam (30 μg), ampicillin (10 μg), and ampicillin-sulbactam (10/10 μg).

### Detection of antibiotic resistance genes

DNA was extracted by the boiling method using TE buffer as previously described [17]. All 94 ESBLs isolates were screened for class A ESBLs (*bla*_GES_, *bla*_SHV_, *bla*_CTX-M_, *bla*_VEB_, *bla*_PER_, and *bla*_TEM_) and class D ESBL (*bla*_OXA-10_) using PCR. In addition, the type of clone O25b/ST131 was detected by confirming the presence of the *pabB* gene [18, 19]. All the primers for the mentioned β-lactamase genes are listed in Table 1.

### Biofilm formation assay and quantification

The biofilm formation was performed in a 96-well polystyrene plate containing 10 O25b/ST131 positive strains, 10 randomly selected negative O25b/ST131 strains and the *E.coli* ATCC 25922 as control. Isolates were incubated overnight in Tryptic Soy Broth (TSB) media (Conda lab, Spain) and then the optical density (OD) of each isolate was adjusted between 0.4 and 0.6 at 600 nm. Furthermore, 190 µl of TSB broth containing of 10 µl of bacterial suspension was added to each well. Incubation was performed overnight at 37 ℃ with continuous shaking at 30 rpm. Biofilm assay for each isolates was performed in triplicate using TSB broth as the negative control. Moreover, after incubation the wells were washed with distilled water, stained with 0.1% crystal violet, and left at room temperature 10 min. After incubation, wells were washed 3 times with distilled water. Eventually, 200 µl of 95% ethanol was added to wells, and the OD was measured at 492 nm using an ELISA reader had measured. The OD values were considered as an index of biofilm formation. Quantitative analysis to evaluate the biofilm formation was performed by calculating the average absorbance of the control wells (Ac) that subtracted from the A492 nm of all test wells. Mean values and standard deviations were calculated for all experiments. Isolates characterized as (4 × Ac) < A = strong biofilm producer, (2 × Ac) < A ≤ (4 × Ac) = moderate biofilm producer, Ac < A ≤ (2 × Ac) = weak biofilm producer and A ≤ Ac = no biofilm producer [20, 21].

### ERIC-PCR typing

ERIC-PCR was performed to evaluate the genetic relationship between ESBLs isolates. Each PCR reaction mixture in a total volume of 20 µL contained: 1 μl of each primer, 10 μl of the master mix (Ampliqon, Denmark), 3.5 μl of template DNA, and 4.5 μl of deionized water. The reaction was as follows: initial denaturation at 94 °C for 1 min, with the 30 cycles, denaturation step at 94 °C for 30 s, annealing at 52 °C for 35 s, extension at 72 °C for 4 min, and final extension for 5 min at 72 °C. The amplicon was electrophoresed on a 1.2–1.5% (w/v) agarose gel containing a safe stain (Yekta Tajhiz Azma, Iran) at 90 V for 90 min. The [DM2100] ExcelBand 100 bp DNA Ladder (Smobio, Taiwan) were used as marker [22, 23].

To calculate the placement and visibility of the gels were assessed by ERIC-PCR according to their molecular weights and molecular markers. Electrophoretic patterns were calculated using BioNumerics gel analysis software (Applied Maths, Belgium). Gel-to-gel banding pattern comparison was performed, to ensure adequateness; the analysis contains a normalization step, that makes each lane an equal length. The “band scoring” process identifies bands of each lane that combined to generate the fingerprint-based on the threshold of stringency and optimization settings, set at 1.0%. By using the Bionumerics, the design of a phylogenetic tree for isolated strains was performed via the presence of a broad range of genetic heterogeneities among their populations. The cut-off for cluster definition was 50%.

### Statistical analysis

All of *E. coli* isolates data were collected and entered into SPSS software, v. 22.0 (SPSS inc., USA) for analysis. Interpretation of demographic information was based on frequency. The association between different genes, antibiotic resistance, and O25b/ST131 clone type were evaluated by using the chi-square (χ^2^) test. The eta (η) correlation ratio was determined to investigate the association between the O25b/ST131 clone type and the level of biofilm formation. The level of statistical significance was set at p ≤ 0.05.

## Results

### Antibiotic susceptibility and resistance determinants

In this study, among 173 *E. coli* isolated from UTI, ESBL-producer isolates were included in the study for further testing. Of the 173 isolates causing UTI, 94 (54.3%) isolates were resistant to one of four-generation of cephalosporins. Of 94 isolates, 35 (37.2%) were isolated from men and 59 (62.8%) were isolated from women. The highest resistance in all isolates was observed to ampicillin (97.9%). Most of the tested antibiotics had susceptibility rate between 1 and 10.6% also, highest susceptibility observed in ceftazidime, cefepime and aztreonam with rates of 72.3%, 52.1% and 47.9%, respectively. Resistance to cefazolin as the first generation of cephalosporin cefuroxime as the second generation was 93.6% and 90.4%, respectively. Although 87.2% and 90.4% of the isolates were resistant to ceftriaxone and cefotaxime, respectively, as the third generation of cephalosporins, only 25.5% of the isolates were resistant to ceftazidime. Resistance to cefepime, a fourth-generation cephalosporin, was also confirmed in 47.9% of isolates. Details of the antibiotic susceptibility test can be found in Table 2.

**Table 2:** Antibiotics resistance pattern in ESBLs and O25b/ST131 clone type isolates of *E. coli*

Antibiotics	ESBLs samples N = 94			O25b/ST131 samples N = 79		
	Resistance	Intermediate	Sensitive	Resistance	Intermediate	Sensitive
Ampicillin	92 (97.9%)	1 (1.1%)	1 (1.1%)	77 (97.4%)	1 (1.2%)	1 (1.2%)
Ampicillin-sulbactam	87 (92.6%)	1 (1.1%)	6 (6.4%)	72 (91.1%)	1 (1.2%)	6 (7.5%)
Aztreonam	48 (51.1%)	1 (1.1%)	45 (47.9%)	42 (53.1%)	1 (1.2%)	36 (45.5%)
Cefazolin	88 (93.6%)	0 (0.0%)	6 (6.4%)	75 (94.9%)	0 (0.0%)	4 (5%)
Cefuroxime	85 (90.4%)	0 (0.0%)	9 (9.6%)	73 (92.4%)	0 (0.0%)	6 (7.5%)
Ceftriaxone	82 (87.2%)	2 (2.1%)	10 (10.6%)	71 (89.8%)	1 (1.2%)	7 (8.8%)
Cefotaxime	85 (90.4%)	2 (2.1%)	7 (7.4%)	72 (91.1%)	1 (1.2%)	6 (7.5%)
Ceftazidime	24 (25.5%)	2 (2.1%)	68 (72.3%)	22 (27.8%)	2 (2.5%)	55 (69.6%)
Cefepime	45 (47.9%)	0 (0.0%)	49 (52.1%)	39 (49.3%)	0 (0.0%)	40 (50.6%)

### Prevalence of O25b/ST131 clonal group and ESBLs-encoding genes

Based on the fact that 94 isolates were resistant to one of the cephalosporins, they underwent ESBL production identification test, which revealed that all of these isolates were phenotypically ESBL-producing. Molecular technique for determining the presence of the ESBL genes showed that 52 (55.3%), 79 (84%), 89 (94.6%), 20 (21.3%), 19 (20.2%), 83 (88.3%), and 4 (4.3%) isolates harbored of *bla*_TEM_*, bla*_SHV_, *bla*-_CTX-M-1_, *bla*-_CTX-M-2_, *bla*-_CTX-M-9_, *bla*-_CTX-M-15,_ and *bla*_PER,_ genes, respectively. On the other hand, *bla-*_CTX-M-8_*, bla-*_CTX-M-25_, and *bla*_VEB_ genes were not found in any of the isolates. Also, the study of *bla*_GES_ and *bla*_OXA_ genes, revealed that none of the isolates carrying these two carbapenemase genes. Using a special primer (pabB) 79 (84%) isolates were determined to belong to the O25b/ST131 clone type.

### Biofilm formation assay

Six out of 10 *E. coli* O25b/ST131 isolates, had ability to form a biofilm, of which 3, 2, and 1 isolates could form strong, moderate, and weak biofilms, respectively. In addition, the weak relationship between O25b/ST131 clone type isolates and the level of the biofilm formation was observed (Eta = 0.243).

### Statistical analysis

**Fig. 1:**
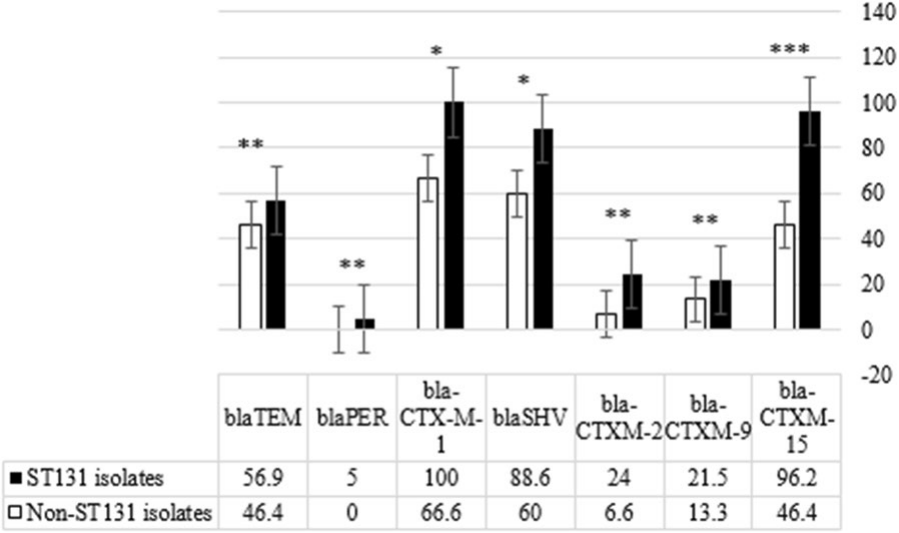
Comparison of the percentage of presence of resistance genes between O25b/ST131 clone type isolates and non-O25b/ST131 isolates. ^*^P-value less than 0.05 = ^*^, less than 0.005 = ^**^, and less than 0.0005 = ^***^

Analysis of antibiotic susceptibility test data showed that, with the exception of ampicillin and ampicillin-sulbactam, the O25b/ST131 clone type isolates had a higher rate of resistance to other antibiotics than the ESBLs samples (Table 2). In addition, it was found that the percentage of resistance genes in O25b/ST131 clone type isolates was higher than in non-O25b/ST131 isolates, and statistical analysis of the data showed a significant association between the presence of *bla-*_CTX-M-1_, *bla-*_CTX-M-2_, *bla-*_CTX-M-9_, *bla*_-CTX-M-15_, *bla*_PER_, genes and O25b/ST131 clone type isolates (P < 0.05). Figure 1 details of the percentage of resistance genes present in O25b/ST131 clone type isolates compared to non-O25b/ST131 isolates.

### ERIC-PCR profiles of ESBLs isolates

**Fig. 2:**
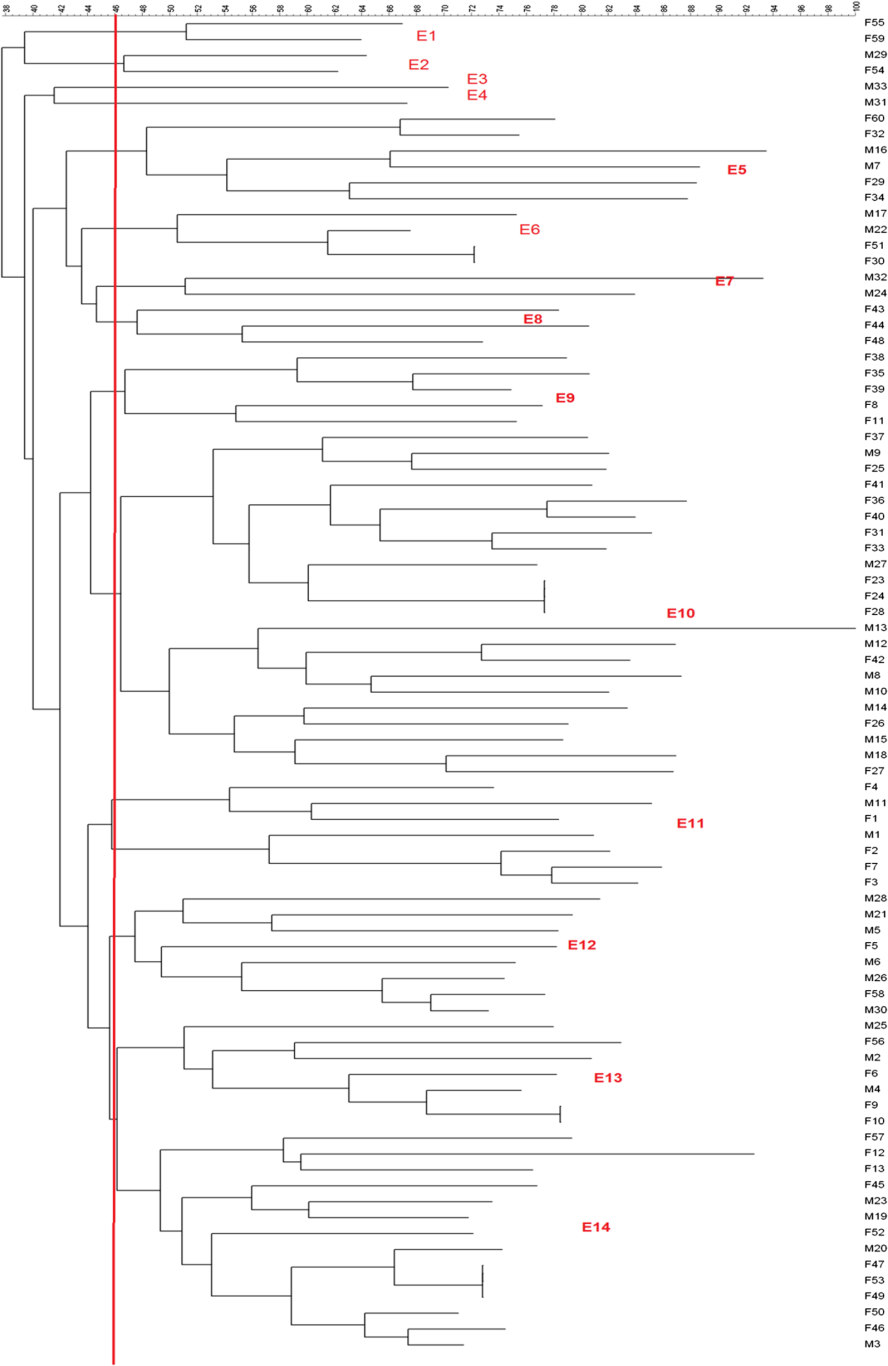
Dendrogram created from enterobacterial repetitive intergenic consensus-polymerase chain reaction (ERIC-PCR) banding pattern of O25b/ST131 isolates from patients with urinary tract infection

Bands were calculated for each sample and a high genetic diversity of *E.coli* was found. The genotyping profiles of 84 ESBL *E. coli* strains in accordance with ERIC-PCR fingerprinting is shown in Fig. 2, and fourteen groups were formed using ERIC-PCR fingerprinting, but 10 strains were not typable. In the studied strains, 22, belonged to the E10 cluster, and the minimum, 1, belonged to the E3 and E4 cluster. Two of the strains were in the E1, E2, and E7. Fourteen were in the E14, 6 strains in the E5, 8 strains in the E12, 3 strains in the E8, 5 strains in the E9, 7 strains in the E11 and the E13 cluster. The predominant DNA fingerprints fragments was identified with the size 750 bp, which was found in 70 strains, and the least frequent, a size of 170 bp, was observed in 2 strains. ERIC-PCR demonstrated that the isolates investigated in the this study had a wide range of genetic diversities and this method showed a good sensitivity in detecting slight differences between isolates. Studies and comparisons of dendrograms and antibiotic susceptibility test results have provided valuable results; i.e., samples in the E10 cluster were sensitive to ceftazidime, cefepime, and aztreonam. The ERIC-PCR banding patterns have shown 0 to 30 bands encompassing 150 bp to 3000 bp.

## Discussion

The number of ESBL-producing bacteria has increased over the past decade, and to control the infections and selection of the most suitable antibiotics showed the importance of detection of these isolates. Furthermore, new policies are required to restrict these isolates spread, especially in a hospital environment [30, 33]. Until the 1990s, the ESBL genes were mostly detected in *Klebsiella pneumoniae* rather than *E. coli* but, in recent years, it has been mainly found in *E. coli* isolates [34]. Previous studies also a study Iran report well-establish relation between ST131 and ESBL production [35, 36] therefore, this study designed to investigate the prevalence of ESBL-producing bacteria belonged to O25b/ST131 clone type among clinical isolates collected from two selective hospitals in Tehran. Of 173 isolates, 94 (54.3%) carried the ESBL genes with the most detected gene (94.6%) being *bla*_-CTX-M-1_. In the current study, antimicrobial resistance was more frequent in the O25b/ST131 clones than non-O25b/ST131 isolates and high resistance to all four generations of cephalosporins was detected. Furthermore, two studies in the United Kingdom and Iran reported the same cephalosporins with resistance rates of 68% and 49.5%, respectively [37, 38]. Moreover, there are several reports in recent years pointed out the importance of ST131 as a major clone for extraintestinal *E. coli* infections [8, 39–41].

Johnson et al. reported that 42.51% of investigated *E. coli* isolates were ST131, of which 67–69% were resistant to extended-spectrum cephalosporins or fluoroquinolones [39]. Furthermore, our study demonstrated 84% of isolates belonged to O25b/ST131, and 89.8% were resistant to extended-spectrum cephalosporins. In addition, other studies confirmed the close association between ST131 and ESBL production also, a recent meta-analysis study demonstrated the high prevalence of broadly disseminated ST131 clone among ESBLs isolates in the western Asia region. Additionally, Iran reported with highest MDR-ST131 isolates in this region, which is similar to our results [35, 36, 42].

The frequency of ESBLs genes, especially the *bla*_CTX-M-15_ has posed a serious threat to public health [43, 44]. Our result indicated that the frequency of ESBLs genes was higher in O25b/ST131 clone type than non-O25b/ST131 isolates, especially in *bla*_CTX-M_ genes. ST131 clone type is known worldwide for its role in the dissemination of ESBLs genes, especially *bla*_CTX-M-15_ [36].

Shin et al. reported that the existence of the plasmid harboring *bla*_CTX-M_ could be a major factor related to the emerging and dissemination of pandemic multi-resistant *E. coli* ST131. In addition, they demonstrated that isolates with plasmids harboring *bla*_CTX-M-14_ or *bla*_CTX-M-15_ showed raise to cephalosporin MICs, in comparison to susceptible hosts. Additionally, they showed high MICs of ampicillin, aztreonam, gentamicin, and piperacillin/tazobactam [45].

Our study showed similar results with the prevalence of *bla*_CTX-M-15_ was 96.2% in O25b/ST131 clone type isolates and high resistance to ceftriaxone, cefotaxime, cefuroxime, cefazolin and ampicillin was observed. Furthermore, the similar study conducted in Iran reports a 95.5% prevalence of *bla*_CTX-M-15_ gene among ESBL positive O25b/ST131 isolates [46].

In addition, a significant prevalence difference in other CTX genes between O25b/ST131 and non-O25b/ST131 isolates was observed. This result is in correlation with the Overdevest et al. study supporting the idea of O25/ST131 success are associated with the ESBL phenotype [47]. Moreover, we selected 10 O25b/ST131 clone type isolates and 10 non-O25b/ST131 and although in vitro biofilm formation is strongly depending on the method, there was a weak association between O25b/ST131 clone type isolates and the level of the biofilm formation, which is correlated with other studies that confirmed the ST131 isolates carrying *bla*_CTX-M-15_ were unable to develop mature biofilm [48].

For genotyping of isolates, ERIC-PCR method was considered with 50% cut-off for cluster definition, which is faster and more cost-effective rather than other techniques. Also, ERIC-PCR showed a higher discriminatory ability in comparison to other quick-typing techniques [30, 49]. As a result of genotyping, the *E. coli* ST131 isolate was genetically diverse and heterogeneous expected; since the isolates were collected from two different hospitals over a 9 months. We detected 14 groups of *E. coli* from 84 isolates and the most of isolates were classified into 6 groups that showed similar profiles which can be explained as the clonal transmission of our isolates. Similarly, another study from Iran reported the high diversity among 230 *E. coli* isolates, collected from two selective hospitals [50].

This study has several limitations including, the isolates being collected from one city rather than the whole country, which limit the generalizability of our data. On the other, more multicenter study are needed in the future to determine the other features of this clone type. The suitable genotyping method is MLST and could be the next step in near future.

In summary, the current study demonstrated once again the importance of *E. coli* O25b/ST131 clone as a type of clone capable of rapidly spreading and disseminate antibiotic resistance genes. Moreover, the study of various mechanisms in this clone type is useful to prevent the transmission of antimicrobial resistance genes, especially with the increasing rate of resistance to colistin among this clone [51]. Our study detected O25b/ST131 with a high resistance rate among clinical isolates of *E. coli* and ESBLs genes among them.

## Data Availability

All data were included.
